# Modulation of Synovial Fluid-Derived Mesenchymal Stem Cells by Intra-Articular and Intraosseous Platelet Rich Plasma Administration

**DOI:** 10.1155/2016/1247950

**Published:** 2016-10-12

**Authors:** Emma Muiños-López, Diego Delgado, Pello Sánchez, Bruno Paiva, Eduardo Anitua, Nicolás Fiz, Beatriz Aizpurua, Jorge Guadilla, Sabino Padilla, Froilán Granero-Moltó, Felipe Prósper, Mikel Sánchez

**Affiliations:** ^1^Cell Therapy Area, Clínica Universidad de Navarra, Av. de Pío XII 36, 31008 Pamplona, Spain; ^2^Arthroscopic Surgery Unit Research, Hospital Vithas San Jose, C/Beato Tomás de Zumarraga 10, 01008 Vitoria-Gasteiz, Spain; ^3^Center for Applied Medical Research, Av. de Pío XII 55, 31008 Pamplona, Spain; ^4^Fundacion Eduardo Anitua, C/Jose María Cagigal 19, 01007 Vitoria-Gasteiz, Spain; ^5^Arthroscopic Surgery Unit, Hospital Vithas San Jose, C/Beato Tomás de Zumarraga 10, 01008 Vitoria-Gasteiz, Spain; ^6^Orthopaedic Surgery and Traumatology Department, Clínica Universidad de Navarra, Av. de Pío XII 36, 31008 Pamplona, Spain; ^7^Hematology Department, Clínica Universidad de Navarra, Av. de Pío XII 36, 31008 Pamplona, Spain

## Abstract

The aim of this study was to evaluate the effect of intra-articular (IA) or a combination of intra-articular and intraosseous (IO) infiltration of Platelet Rich Plasma (PRP) on the cellular content of synovial fluid (SF) of osteoarthritic patients. Thirty-one patients received a single infiltration of PRP either in the IA space (*n* = 14) or in the IA space together with two IO infiltrations, one in the medial femoral condyle and one in the tibial plateau (*n* = 17). SF was collected before and after one week of the infiltration. The presence in the SF of mesenchymal stem cells (MSCs), monocytes, and lymphocytes was determined and quantified by flow cytometry. The number and identity of the MSCs were further confirmed by colony-forming and differentiation assays. PRP infiltration into the subchondral bone (SB) and the IA space induced a reduction in the population of MSCs in the SF. This reduction in MSCs was further confirmed by colony-forming (CFU-F) assay. On the contrary, IA infiltration alone did not cause variations in any of the cellular populations by flow cytometry or CFU-F assay. The SF of osteoarthritic patients contains a population of MSCs that can be modulated by PRP infiltration of the SB compartment.

## 1. Introduction

Knee osteoarthritis (OA) encompasses a cluster of degenerative joint conditions with different biochemical, inflammatory, and genetic signatures generating distinct subtypes. Evolving in phases, the severity of the resulting phenotype impacts the quality of life of the patient and represents an economic burden and social challenge. Estimates suggest that about 46 million patients suffer from OA in developed countries, more than 50% of adults over 50 years; by 2030, this figure may reach 70 million [1]. It is essential to develop novel treatments that slow or stop the progression of this disease and even reverse the damage. Current treatments such as analgesics, nonsteroidal anti-inflammatory drugs, intra-articular infiltrations of steroids, or hyaluronic acid just relieve the symptoms, and, in advanced cases of OA, joint replacement is the only solution for these patients [2].

The knee joint is a complex biological system composed of synovial fluid (SF), synovial membrane (SM), meniscus, ligaments, subchondral bone (SB), and articular cartilage (AC). AC is an avascular tissue that lies functionally sandwiched between the SM, which generates the SF, and the SB. Stemming primarily from an ultrafiltrate of plasma and secretions of chondrocytes and synoviocytes, SF is a viscous liquid composed of hyaluronan (HA) and lubricin, cytokines, growth factors, and a minor presence of cells. Aggression and inflammation to intra-articular tissues bring an increase of MSCs in SF [3, 4], which is commonly interpreted as a tissue response to injury [5, 6], equivalent to the response of migratory chondrogenic progenitor cells from SB to injured cartilage [7, 8]. Although the source of MSCs has not been yet clearly determined, the most likely origin might be the SM [4, 5], the breakdown zone of superficial AC, and the SB [6, 9, 10]. Recent findings suggest that the increase in pathological situations of certain molecules such as monocyte chemotactic protein-1, SDF-1, and TGF-*β*1 could promote the recruitment of MSCs [11, 12].

SB has always been present in the equation of OA pathogenesis [13]. There is an increasingly recognized communication between the SB and AC based on the changes that the SB undergoes in patients with severe OA, including microcracks and structural defects, vascularization of channels, nerve growth, and a progressive replacement of the subchondral marrow with fibroneurovascular mesenchymal tissue [9, 10, 14, 15]. Since the primary driver of knee OA is not yet established between the different joint tissues, therapeutic strategies solely targeting one cell or tissue are prone to fail [16]. Thus, approaches to treat OA should be aimed at reaching several joint tissues with the purpose of reducing joint inflammation, controlling pain, improving joint functionality, and restoring tissue homeostasis.

Among the new emerging treatments to address knee OA, mesenchymal stem cells (MSCs) and Platelet Rich Plasma (PRP) stand out [17], with the scientific rationale for the use of PRP in the treatment of knee OA growing. Intra-articular infiltrations of PRP have proven to substantially reduce pain in patients with knee and hip OA and to improve joint stiffness and physical function [18–21]. PRP and many of the bioactive mediators that contain IGF-I, TGF-*β*1, HGF, PDGF, VEGF, NGF, BDNF, CTGF, BMPs, Vitronectin, fibronectin, SDF-1, and PF4, among others, have shown positive effects on homeostasis of joint tissues through chondroprotective, anabolic, anti-inflammatory, and immunomodulatory effects [22–26]. Also MSCs hold an important therapeutic potential promoting regeneration, derived from their proliferative and multipotential differentiation properties. MSCs could lead to the formation of new chondrocytes and cartilage regeneration, a process that has been observed in promising preclinical studies and clinical trials [27–29]. However, there are still specificities on this broader treatment that require deeper analysis as to what cell sources are more appropriate, influence on therapeutic effectiveness of* in vitro* expansion, and dosage [30]. We hypothesize that targeting SM, SF, AC, and SB with a combination of intra-articular injections and intraosseous (IO) infiltrations of PRP on severe knee OA [31] could have a deeper biological impact on knee joints tissues and therefore be a more effective treatment than the conventional intra-articular (IA) infiltrations of PRP.

## 2. Methods

### 2.1. Treatment Groups and Collection of Synovial Fluids

Patients were divided into two modality treatment groups; patients of the IA modality group received a single IA infiltration of PRP (*n* = 14) and patients of the IO group (*n* = 17) were treated with a combination of one IA infiltration of PRP followed by two PRP IO infiltrations of PRP (one in the tibial plateau and one in the medial femoral condyle). Both groups received two more IA infiltrations of PRP on a weekly basis. SF were collected from 31 patients, before and after the first week of PRP treatment. The choice of IA or IO modality treatment was made based on the failure of previous medical treatments; namely, the patients who had been oriented toward a total knee replacement as the only solution for their OA were allocated in the IO group.

### 2.2. PRP Preparation

A small volume between 36 and 72 mL of peripheral blood was extracted from each patient into extraction tubes containing 3.8% sodium citrate as anticoagulant. After centrifugation at 580 ×g for 8 minutes, plasma fractions were separated by pipetting under sterile conditions. In each tube, the 2 mL of plasma rich in platelets remaining above the red cells and the “buffy coat” were collected, avoiding picking up the leukocytes, and were put together [31]. This preparation was characterized by containing 2 to 3 times the concentration of platelets compared with peripheral blood and the absence of erythrocytes and leukocytes (BTI Biotechnology Institute, Vitoria-Gasteiz, Spain).

### 2.3. Procedures

For the IA group, 8 mL of PRP was infiltrated in the joint space. Before infiltration, a 21 G needle was placed into the joint space and SF arthrocentesis was carried out and collected SF were preserved for analysis as pretreatment sample. One week after, another arthrocentesis was carried out to analyze the SF after treatment. For the IO group, a sedation of the patient was induced by infusing a single dose of normal saline, a single dose of midazolam (0.03–0.05 mg/kg), and fentanyl (3.2 mg/kg), in peripheral vein; single or repeated dose of propofol was also administered (1-2 mg/kg), dependent on the duration of the infiltration. The degree of sedation was −4 or −5 on the Richmond Sedation Scale. Local anesthesia was conducted by injecting 2 mL of 2% mepivacaine into the periosteum of the condyle and tibial plateau. As in the case of IA group, an arthrocentesis was carried out to evacuate the totality of SF which was preserved for analysis as pretreatment sample of IO group. PRP was infiltrated into the joint space first (8 mL) and then into SB of the tibial plateau (5 mL) and the femoral condyle (5 mL), using a 13 G bone-biopsy trocar manually introduced into SB; the use of the fluoroscope facilitated trocar placement. It is worth noting that Sánchez et al. have illustrated visual direct evidence that the intraosseously injected PRP was allocated into the SB [32].

The institutional review board approved this study, and informed consents were obtained from every patient included in the study.

### 2.4. Multidimensional Flow Cytometry (MFC) Immunophenotyping

**Figure 1:**
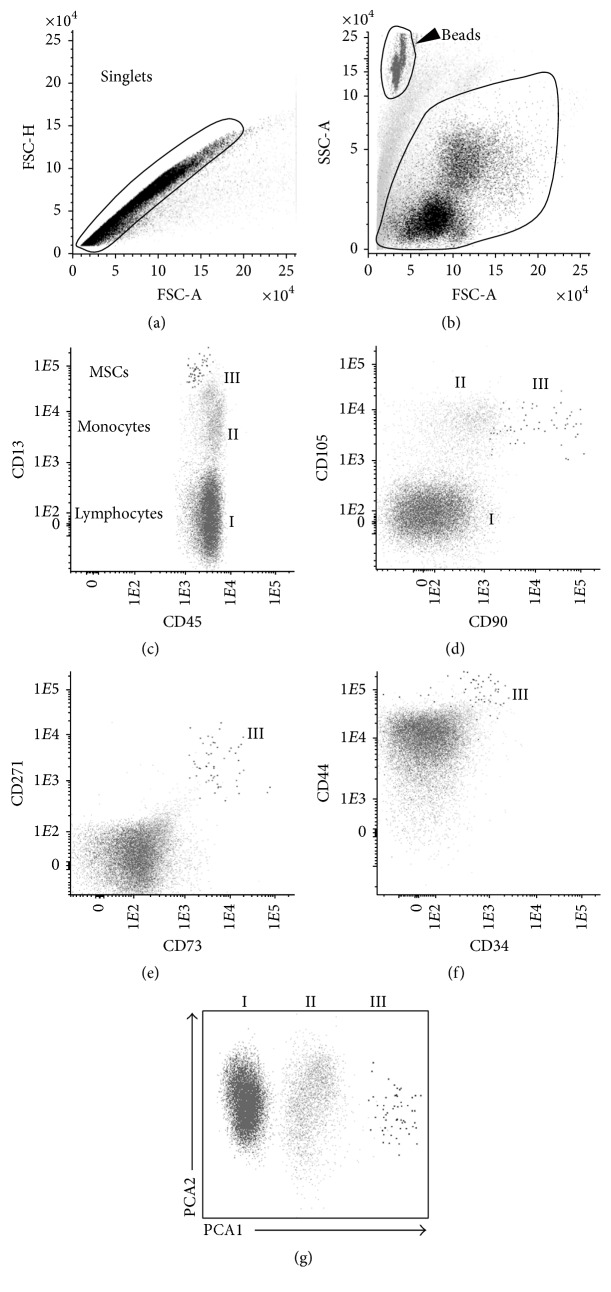
Phenotypic characterization of synovial fluid MSCs. After exclusion of doublets (a) and debris (b), mesenchymal stem cells (MSCs) were identified through a Boolean gating strategy according to their strong reactivity for CD13, CD44, CD73, CD90, and CD105 and intermediate to high levels of CD271 (e-f), in the absence of CD34 (f). Monocytes were defined on the basis of their relatively higher light scatter properties and CD13 and CD45 bright expression, whereas lymphocytes were identified through low scatter properties and strong CD45 reactivity. In panel (g), the automated population separator (APS) graphic representation of the Infinicyt software is shown with the three cell populations phenotypically separated by principal component analysis (PCA). I: lymphocytes; II: monocytes; III: MSCs.

Approximately 2–6 mL of arthrocentesis-derived SF of each patient was immunophenotyped using an 8-color direct immunofluorescence technique. After sample centrifugation, 100 *μ*L of the concentrated cell suspension was stained for 15 minutes at room temperature in darkness, with the following combination of monoclonal antibodies (MoAb): Brilliant violet (BV) 421/orange chrome (OC) 500/fluorescein isothiocyanate (FITC)/phycoerythrin (PE)/peridinin chlorophyll protein-cyanin 5.5 (PerCP-Cy5.5)/PE-cyanin 7 (PE-Cy7)/allophycocyanin (APC)/APCH7: (i) CD105/CD45/CD73/CD271/CD34/CD13/CD90/CD44. After staining, 2 mL of FACS lysing solution (Becton/Dickinson Biosciences, San Jose, CA) was added. After 5 minutes of incubation at room temperature, samples were sequentially centrifuged for 5 minutes at 540 ×g and resuspended in 100 *μ*L of premixed Perfect-COUNT microspheres (Cytognos SL, Salamanca, Spain). Subsequently, data acquisition was performed for around 5,000 nucleated cells per tube in a FACSCantoII flow cytometer (Becton Dickinson Biosciences (BD), San Jose, CA) using the FACSDiva 6.1 software (BD). Monitoring of instrument performance was performed daily using the Cytometer Setup Tracking (CST; BD) and rainbow 8-peak beads (Spherotech Inc., Lake Forest, IL) after laser stabilization, following the EuroFlow guidelines; sample acquisition was systematically performed after longitudinal instrument stability was confirmed. MSCs and residual leukocytes were identified through a Boolean gating strategy based on forward scatter, side scatter, and CD45 expression; monocytes were defined on the basis of their relatively higher light scatter properties and CD13 and CD45 bright expression, whereas lymphocytes were identified through low scatter properties and strong CD45 reactivity (Figure 1). Absolute cell numbers per volume unit were calculated following the manufacturer's recommendation.

### 2.5. MSCs Isolation from Knee Synovial Fluid

Collected SF were diluted in phosphate buffer saline (PBS) and the cellular content was then harvested by centrifugation. One part of each sample was seeded in a 6-well plate under standard cell culture conditions with Dulbecco's Modified Eagle Medium (DMEM; Lonza) supplemented with 20% fetal bovine serum (Gibco), 1% penicillin-streptomycin (P/E) (Gibco), and 1 ng/mL of human recombinant basic fibroblast growth factor (bFGF; R&D systems) (Expansion Medium). The adherent cells were expanded in a humidified 5% CO_2_ atmosphere at 37°C and used for further differentiation experiments. The remaining sample was used for colony-forming assay (CFU-F) and seeded on a 100 mm diameter culture plate. Seven days later, plating colonies were visible and counted by 0.5% crystal violet staining. It was established that a CFU-F contains more than 10 morphologically homogeneous cells.

### 2.6. Synovial Fluid MSCs Differentiation

Mesenchymal lineage differentiation assays were carried out as described in Muinos-López et al. 2016 [33]. Briefly, SF-derived cells were assessed between passages 2 and 5 to confirm their osteogenic, adipogenic, and chondrogenic capacity. For osteogenic and adipogenic differentiation, 8000 cells/cm^2^ were seeded in 12-well plates. Adipogenic differentiation was induced using DMEM supplemented with 10% FBS, 1 *μ*M Dexamethasone, 0.5 mM 3-isobutyl-1-methylxanthine, and 50 *μ*M Indomethacin for 21 days. For the osteogenic differentiation, cells were cultured in DMEM supplemented with 10% FBS, 50 *μ*g/mL L-(+)-ascorbic acid, 10 mM *β*-glycerol phosphate, and 10 nM Dexamethasone for 21 days. For chondrogenic differentiation, 2.5*E*5 cells were spun-down at 600 ×g for 10 minutes in polystyrene 15 mL conical tubes and incubated with hMSC Chondrogenic Differentiation BulletKit™ Medium (Lonza). Differentiations were analyzed at 28 days. In all differentiation assays, a negative control was included where the cells were maintained with expansion medium (DMEM containing 10% FBS) without induction factors. In all differentiation assays, medium was changed every 2-3 days.

### 2.7. Histological and Immunohistochemistry Differentiation Analyses

Adipogenic and osteogenic differentiation were assessed by Oil Red O and Alizarin Red staining, respectively. For adipogenic differentiation, after fixation with 4% paraformaldehyde (Panreac) for 10 minutes, cells were rinsed with 60% isopropyl alcohol followed by a 60% solution of Oil Red for 20 minutes to reveal intracellular oil droplets. For osteogenic differentiation, mineral precipitates were revealed with a 2% solution of Alizarin Red, pH 4.2, for 15 minutes at room temperature and washed with deionized water. Chondrogenic differentiation was evaluated by toluidine blue staining and immunohistochemistry (IHC) for type II collagen. Cell pellets were included in paraffin and sectioned, 4 *μ*m thick. Toluidine Blue, 1% (weight/volume) in 1% acetic acid solution, was used to visualize anionic glycoconjugates, proteoglycans (PG), and glycosaminoglycans (GAG). For IHC, sections were hydrated in grade ethanol and subjected to antigen unmasking by sequential 15 min treatments of hyaluronidase (4 mg/mL in PBS) and pepsin (4 mg/mL in 0.01 N HCl solution) at 37°C. Endogenous peroxidase activity was blocked by H_2_O_2_ treatment (3% H_2_O_2_ in PBS). Samples were incubated overnight at 4°C with a mouse monoclonal antihuman type II collagen (0.5 *μ*g/mL; Clone II-4CII, MP Biomedicals). Staining was visualized with DAB using EnVision® chromogenic kit (DAKO) according to the manufacturer's instructions.

### 2.8. Statistical Analysis

Data were determined by the mean and standard deviation. Comparisons were performed by Wilcoxon signed-rank test for nonparametric data and Student's *t*-test for parametric data, after assessing the normal distribution of the samples by Shapiro-Wilk test. Data were considered statistically significant when *p* values were less than 0.05. Statistical analysis was performed with SPSS 17.0 (SPSS, Chicago, IL).

## 3. Results

### 3.1. Characteristics of the Patients

The mean age of patients in the IA group was 62.6 ± 11.8 years and the range was 41–77 years. The percentages of patients of this group with osteoarthritis grades II, III, and IV according to Ahlbäck scale were 50%, 35.7%, and 14.3%, respectively. Regarding the IO group, the average age of patients was 63.6 ± 11.2 years and the range was 41–80 years. In this group, the percentages of patients classified by Ahlbäck scale were 29.4% for grade II, 47.1% for grade III, and 23.5% for grade IV (Table 1).

### 3.2. Phenotypic Characterization of the Cell Population of Synovial Fluid

To determine the influence of PRP treatment in the cellularity of the joint, the presence of mononucleated cells (MNC) cells and their populations was analyzed in the SF of both groups, before and after treatment, by flow cytometry, as described in Methods (Figure 1).

Regarding the IA group, the concentration of MNC, lymphocytes, monocytes, and MSCs in the SF before and after treatment did not show significant differences (Table 2).

Interestingly, although in the IO group the variations in the concentration of MNC, lymphocytes, and monocytes in the SF were also not significant, MSCs showed a significant decrease after IO treatment (Table 3).

Table 4 shows the cellular increments (*δ*) before and after each infiltration and compares the differences between the two treatments. The decrease in the levels of MSCs observed after IO infiltration of PRP was higher than the decrease after IA treatment (*p* = 0.045).

### 3.3. Culturing of Colony-Forming Cells (CFU-F) 

**Figure 2:**
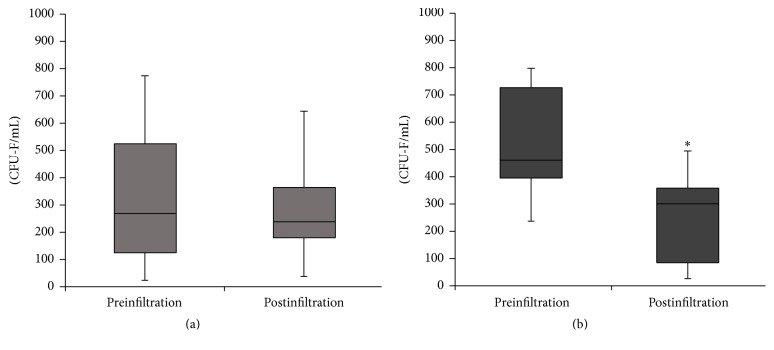
Colony-forming units fibroblast. Levels of colony-forming units fibroblast in the synovial fluids (CFU-F) before (preinfiltration) and one week after (postinfiltration) infiltration of Platelet Rich Plasm (PRP). (a) Intra-articular infiltration of PRP. (b) IO infiltration of PRP. ^*∗*^ *p* < 0.05.

To confirm the reduction of MSCs in the SF, we assessed the capacity of the MSCs population to sustain clonal growth on plastic surfaces (CFU-F). Consistent with the flow cytometry results, the IA injection of PRP did not result in a significant variation in CFU-F, 332.52 ± 234.96 CFU/mL before treatment to 327.54 ± 223.32 CFU/mL after treatment (*p* = 0.92) (Figure 2(a)). In the IO group, we found a significant reduction in CFU-F from 477.51 ± 253.44 CFU/mL before IO injections to 222.95 ± 151.36 CFU/mL one week after infiltration (*p* < 0.01) (Figure 2(b)). Consistent with the results obtained with the number of MSCs, the decrease in the CFU-F levels after IO infiltration was greater than the decrease after IA injection (*p* = 0.037).

**Figure 3:**
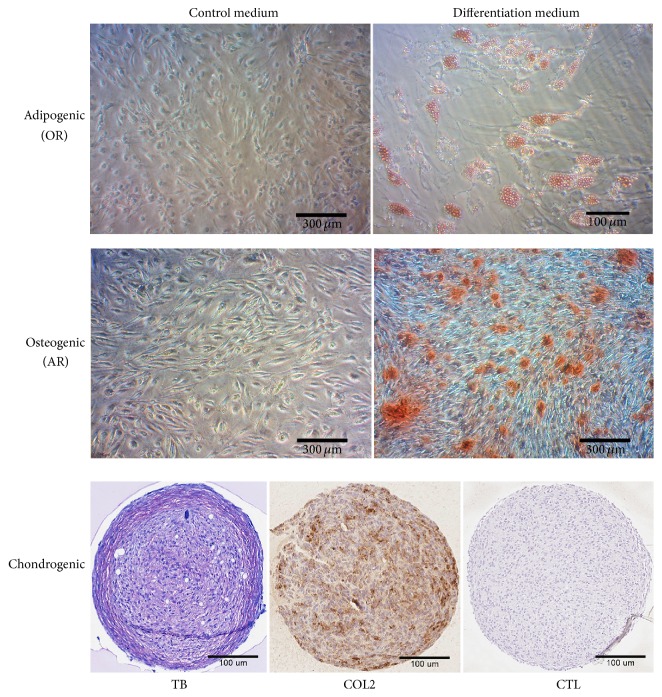
Differentiation assay.* In vitro* differentiation assay of synovial fluid isolated cells to the mesenchymal lineages, adipocytes, osteoblasts, and chondrocytes. Control medium was expansion medium. Adipogenic differentiation was visualized by Oil Red (OR) staining. Osteogenic differentiation was visualized with Alizarin Red (AR) staining. Chondrogenic differentiation was visualized with Toluidine Blue (TB) staining and COL2 and immunohistochemistry using a monoclonal antibody directed to type II collagen. CTL, no primary antibody was added.

To confirm the mesenchymal progenitor nature of the CFU-F cells present in the SF, we performed an* in vitro* multipotency assay by differentiation to the three mesenchymal lineages osteoblast, adipocyte, and chondrocyte under defined conditions (Figure 3). Although only a limited number of assays showed trilineage differentiation capacity (7 out of 68 assays, 10%), the majority of the assessed synovial fluid-derived mesenchymal cells showed bilineage differentiation capacity (51 out of 68, 75%), with a majority of assays positive for adipogenesis and osteogenesis lineage (97%), supporting the mesenchymal nature of the population.

## 4. Discussion

In this study, we carried out two different treatment modalities of PRP applications on OA patients. IA group received intra-articular injections of PRP and a combination of intra-articular and intraosseous injections was applied in the IO group in order to address the SB.

One week after administration of IA infiltration, it was observed that MSCs and monocytes level in SF decreased (Table 2). Although this decrease was not significant, it could suggest an anti-inflammatory effect of PRP. This trend may be more pronounced after two more PRP IA injections, which would be consistent with the significant clinical improvement reported by Sánchez et al. and Vaquerizo et al. using three IA administrations of PRP on a weekly basis [19, 21]. This conventional modality to deliver PRP in patients results in a liquid-to-gel transition 3D fibrin scaffold. When fibrinolysis degrades this scaffold, growth factors within the fibrin scaffold such as IGF-I, HGF, PDGF, TGF-*β*1, and platelet microparticles are released gradually. These growth factors have been proven to promote an anti-inflammatory macrophage phenotype [23, 34–36] and suppress the NF-*κβ* signaling pathway in synovial fibroblasts and chondrocytes of the superficial zone of AC [24] and induce the synthesis of hyaluronic acid and lubricin by synoviocytes and chondrocytes, respectively, with the latter preventing chondrocyte apoptosis, cartilage breakdown, and inhibition of the MSC release and migration [25, 37–39]. Although the decline of monocytes in the SF was not statistically significant, this fact together with all these modulatory and trophic effects of intra-articularly injected PRP on the SM, superficial AC, and SF could suggest a lower level of proinflammatory cytokines and restoration of the joint homeostasis leading to a more favorable SF environment for chondrogenic differentiation of MSCs [30, 37, 39, 40].

Concerning IO group, levels of monocytes and cells also declined, but in this case decrease in the concentration of MSCs was statistically significant (Table 3). This was also confirmed when the levels of CFU-F were analyzed before and after treatment administration (Figure 2(b)). It is worth mentioning that the MSCs population in SF before the PRP treatment and the degree of OA severity was considerably varied between both groups. The levels of SF-MSCs in the IA group were very close to healthy population levels and substantially lower than in the IO group. Likewise, the percentage of patients in the IO group with advanced degree of OA (OA grades III and IV) was 70.6% compared with 50% in the IA group. This difference between the two groups became similar after application of the IO treatment, which approximated the MSCs level to IA group and the healthy population. This observation is in accordance with several studies where the SF-MSCs levels were associated with the severity of OA, joint damage, and the disease duration [4, 34].

When comparing the two treatment groups, the decrease in MSCs after PRP treatment was more pronounced in the IO group (Table 4). Although the drop in the IO group could be influenced by the higher level of MSC present in this group before treatment, this greater decrease was also observed in the CFU-F, where the baseline difference between groups is not so critical. The influence of arthrocentesis in this cell drop must also be taken into consideration, since a week might not be enough for MSCs to migrate to the IA space. Considering that the SM and the breakdown zone of superficial AC are postulated as the main sources of cells that reach the SF and they are continuously soaked with this fluid, it seems possible that MSCs repopulate the SF in a week [4, 5, 41]. Regarding MSCs migration from SB, and despite the lack of clinical studies that analyze the time needed for this process,* in vitro* studies have shown this migration after 20 hours, so a week seems enough for MSCs to reach the SF from SB [42].

This observation suggests that, in the modulation of MSC by PRP, the SB is an important player and potential tissue target and might be a MSC egress point through the channels and vessels breaching the osteochondral junction and reaching the cartilage, partially recruited by the osteoarthritic environment of the SF [9, 10, 42]. The excessive presence of TGF-*β*1 and VEGF in osteoarthritic SB may be a driving factor for changes in osteoblast-osteoclast coupling, which lead to a bone remodeling imbalance and fibroneurovascular growth [9, 10, 12, 16]. Moreover, Zhen et al. showed that by inhibiting TGF-*β* signaling in a specific population of MSCs present at the SB (Nestin positive MSCs) the severity of OA was reduced [12]. In fact, previous studies have shown that the decrease in MSCs in the SF, in low degree OA, suggests clinical improvement [4]. It is reasonable to speculate that, by administering PRP directly into SB, the concurrent presence of platelet-secreted TGF-*β*1 and VEGF as well as plasma growth factors such as IGF-I and HGF could have a modulatory effect on TGF-*β* signaling pathway [12, 43]. This might reduce the presence of MSCs and could likely be associated with the shrinking of fibroneurovascular tissue of OA SB, an explanation which parallels the antifibrotic mechanism already reported in several cell phenotypes [43, 44].

A further significant component to the SF-MSC reduction induced by PRP treatment would be the process of cell* homing* whereby SF-MSCs might be locally recruited to damaged areas of the AC taking part in the* in vivo* repair of this tissue, a possibility already reported by Lee et al. [45]. It has been reported that PRP is rich in fibronectin, a plasma protein incorporated into the fibrin network during the natural polymerization and one of the major factors for the recruitment of mesenchymal progenitor cells [37, 46–48].

Another interesting aspect in our study is to analyze the SF as suitable source of MSCs. Using flow cytometry analysis prior to treatment, the presence of MSCs was observed in the SF in 21 of the 31 enrolled patients, representing 67.7% in total. The level of MSCs in these SF was as low as 5.19 ± 7.15 MSCs/*μ*L. However, the use of this technique to measure fresh SF without a prior cell expansion cycle can represent a limitation due to the low number of cells [35]. In order to overcome this limitation, the presence of MSCs in those SF was evaluated by means of culturing on plastic surfaces to determine the presence of colony-forming cells (CFU-F). In this case, CFU-Fs were found in the SF of all patients, with an average value of 410.59 ± 246.36 CFU-F/mL. These results are consistent with those reported in other studies in which the possibility of using SF as a source of autologous MSCs is demonstrated [5, 34]. This source of cells for obtaining MSCs may be a promising alternative for treating diseases related to cartilage degeneration diseases such as OA.

Various factors must be considered when deciding the cell source and good environmental conditions for optimal effects [30]. The advantage of using SF as a cell source over other niches, such as bone marrow or fat tissue, is foremost its easy access. Arthrocentesis is usually a necessary step prior to conducting an IA injection of corticosteroids, hyaluronic acid, or PRP. Additionally, MSCs present in the SF may derive from the SM, a tissue involved in the cartilage repair process [49, 50], and their chondrogenic capacity could be increased compared with other types of MSCs [51].

This study has some limitations. First, a relatively small number of samples were analyzed and no data were obtained after second and third infiltrations of both treatments because many patients did not present with knee swelling in their last visits. Second, there is a difficulty in working with synovial fluid, for both its complexity and small volumes obtained. Because of this, a cytokine analysis in order to study the inflammatory process could not be carried out, so the work focused mainly on cellularity. Third, the donor-related variability concerning the amount of platelet-derived and plasmatic growth factors present in the PRP could account for the disparity in biological and clinical outcomes.

## 5. Conclusions

In summary, targeting different knee joint structures such as SM, AC, and SB with IA and IO infiltrations of PRP reduces the inflammatory environment and MSCs in SF.* In vitro* differentiation assays for SF-MSCs from OA patients showed different grades of multipotency toward the adipocyte, osteoblast, and chondrocyte lineages, although bilineage differentiation capacity was most frequently observed, confirming their identity. MSC modulation generated by PRP may be increased by acting directly on the SB, whose influence is crucial to the pathogenesis of OA. In addition, the use of PRP may favor MSCs therapeutic effect by decreasing proinflammatory processes present in the SF of OA patients. While being promising, a limitation of our study is the considerable intersubject variability; therefore, a larger sample would possibly be necessary to draw more definitive conclusions. Our results encourage further studies in order to shed more light on the cellular and molecular mechanisms and to elucidate whether the PRP application in both modalities might lead to structural joint tissue changes as* in vitro* and preclinical researches using this therapy have reported [26, 39, 52]. Finally, further studies will be needed in order to increase our knowledge about SF as source of MSC and their therapeutic potential.

## Figures and Tables

**Table 1:** Patients included in the study and their clinical OA grade.

	IA group	IO group
Age (mean ± SD)	62.6 ± 11.8	63.6 ± 11.2
Age range	41–77	41–80
OA grade II (%)	50	29.4
OA grade III (%)	35.7	47.1
OA grade IV (%)	14.3	23.5

**Table 2:** Phenotypic characterization of the cell population in SF of IA group.

	Pretreatment (mean ± SD)	Posttreatment (mean ± SD)	*p* value
MNC (cells/mL)	237.11 ± 223.32	243.81 ± 193.37	0.32
Lymphocytes (cells/mL)	103.65 ± 125.00	85.38 ± 94.16	0.06
Monocytes (cells/mL)	130.66 ± 101.88	142.62 ± 112.81	0.73
MSCs (cells/mL)	2.60 ± 4.38	1.53 ± 2.51	0.32

MNC, mononuclear cells; MSCs, mesenchymal stem cells.

**Table 3:** Phenotypic characterization of the cell populations in SF of IO group.

	Pretreatment (mean ± SD)	Posttreatment (mean ± SD)	*p* value
MNC (cells/mL)	441.92 ± 371.87	354.82 ± 411.44	0.38
Lymphocytes (cells/mL)	179.83 ± 237.87	184.19 ± 337.00	0.072
Monocytes (cells/mL)	199.37 ± 160.28	119.06 ± 98.47	0.053
MSCs (cells/mL)	7.61 ± 8.68	2.46 ± 3.86	0.01

MNC, mononuclear cells; MSCs, mesenchymal stem cells.

**Table 4:** Cellular increment (*δ*).

	IA group (mean ± SD)	IO group (mean ± SD)	*p* value
MNC (cells/mL)	109.70 ± 272.66	−91.33 ± 334.47	0.905
Lymphocytes (cells/mL)	−65.04 ± 106.50	42.64 ± 171.96	0.159
Monocytes (cells/mL)	−19.64 ± 156.00	−97.80 ± 147.95	0.280
MSCs (cells/mL)	−1.41 ± 5.38	−6.36 ± 6.64	0.045
CFU-F (CFU/mL)	−6.87 ± 236.79	−266.30 ± 296.79	0.037

MNC, mononuclear cells; MSCs, mesenchymal stem cells; CFU-F, colony-forming unit fibroblast.

## References

[1] Gabay O. (2012). Osteoarthritis: new perspectives. *Journal of Spine*.

[2] Mobasheri A. (2013). The future of osteoarthritis therapeutics: emerging biological therapy. *Current Rheumatology Reports*.

[3] Matsukura Y., Muneta T., Tsuji K. (2015). Mouse synovial mesenchymal stem cells increase in yield with knee inflammation. *Journal of Orthopaedic Research*.

[4] Sekiya I., Ojima M., Suzuki S. (2012). Human mesenchymal stem cells in synovial fluid increase in the knee with degenerated cartilage and osteoarthritis. *Journal of Orthopaedic Research*.

[5] Jones E. A., Crawford A., English A. (2008). Synovial fluid mesenchymal stem cells in health and early osteoarthritis: detection and functional evaluation at the single-cell level. *Arthritis and Rheumatism*.

[6] Morito T., Muneta T., Hara K. (2008). Synovial fluid-derived mesenchymal stem cells increase after intra-articular ligament injury in humans. *Rheumatology*.

[7] Alsalameh S., Amin R., Gemba T., Lotz M. (2004). Identification of mesenchymal progenitor cells in normal and osteoarthritic human articular cartilage. *Arthritis & Rheumatism*.

[8] Koelling S., Kruegel J., Irmer M. (2009). Migratory chondrogenic progenitor cells from repair tissue during the later stages of human osteoarthritis. *Cell Stem Cell*.

[9] Lajeunesse D., Martel-Pelletier, Pelletier J.-P. (2011). Subchondral bone involvement in the pathophysiology of osteoarthritis. *Understanding Osteoarthritis from Bench to Bedside*.

[10] Suri S., Walsh D. A. (2012). Osteochondral alterations in osteoarthritis. *Bone*.

[11] Campbell T. M., Churchman S. M., Gomez A. (2016). Mesenchymal stem cell alterations in bone marrow lesions in patients with hip osteoarthritis. *Arthritis & Rheumatology*.

[12] Zhen G., Wen C., Jia X. (2013). Inhibition of TGF-*β* signaling in mesenchymal stem cells of subchondral bone attenuates osteoarthritis. *Nature Medicine*.

[13] Radin E. L., Paul I. L., Rose R. M. (1972). Role of mechanical factors in pathogenesis of primary osteoarthritis. *The Lancet*.

[14] Pan J., Wang B., Li W. (2012). Elevated cross-talk between subchondral bone and cartilage in osteoarthritic joints. *Bone*.

[15] Tat S. K., Lajeunesse D., Pelletier J.-P., Martel-Pelletier J. (2010). Targeting subchondral bone for treating osteoarthritis: what is the evidence?. *Best Practice and Research: Clinical Rheumatology*.

[16] Jones E. G. P., Yang X., McGonagle D. (2013). Mesenchymal stem cells and skeletal regeneration. *Mesenchymal Stem Cells and Skeletal Regeneration*.

[17] Roubille C., Pelletier J.-P., Martel-Pelletier J. (2013). New and emerging treatments for osteoarthritis management: will the dream come true with personalized medicine?. *Expert Opinion on Pharmacotherapy*.

[18] Filardo G., Kon E., Pereira Ruiz M. T. (2012). Platelet-rich plasma intra-articular injections for cartilage degeneration and osteoarthritis: single—versus double-spinning approach. *Knee Surgery, Sports Traumatology, Arthroscopy*.

[19] Sánchez M., Fiz N., Azofra J. (2012). A randomized clinical trial evaluating plasma rich in growth factors (PRGF-Endoret) versus hyaluronic acid in the short-term treatment of symptomatic knee osteoarthritis. *Arthroscopy*.

[20] Sánchez M., Guadilla J., Fiz N., Andia I. (2012). Ultrasound-guided platelet-rich plasma injections for the treatment of osteoarthritis of the hip. *Rheumatology*.

[21] Vaquerizo V., Plasencia M. Á., Arribas I. (2013). Comparison of intra-articular injections of plasma rich in growth factors (PRGF-Endoret) versus durolane hyaluronic acid in the treatment of patients with symptomatic osteoarthritis: a randomized controlled trial. *Arthroscopy*.

[22] Anitua E., Sánchez M., Nurden A. T. (2007). Platelet-released growth factors enhance the secretion of hyaluronic acid and induce hepatocyte growth factor production by synovial fibroblasts from arthritic patients. *Rheumatology*.

[23] Coudriet G. M., He J., Trucco M., Mars W. M., Piganelli J. D. (2010). Hepatocyte growth factor modulates interleukin-6 production in bone marrow derived macrophages: implications for inflammatory mediated diseases. *PLoS ONE*.

[24] Montaseri A., Busch F., Mobasheri A. (2011). IGF-1 and PDGF-bb suppress IL-1*β*-induced cartilage degradation through down-regulation of NF-*κ*B signaling: involvement of Src/PI-3k/AKT pathway. *PLoS ONE*.

[25] Sakata R., McNary S. M., Miyatake K. (2015). Stimulation of the superficial zone protein and lubrication in the articular cartilage by human platelet-rich plasma. *The American Journal of Sports Medicine*.

[26] Wu C.-C., Chen W.-H., Zao B. (2011). Regenerative potentials of platelet-rich plasma enhanced by collagen in retrieving pro-inflammatory cytokine-inhibited chondrogenesis. *Biomaterials*.

[27] Filardo G., Madry H., Jelic M., Roffi A., Cucchiarini M., Kon E. (2013). Mesenchymal stem cells for the treatment of cartilage lesions: from preclinical findings to clinical application in orthopaedics. *Knee Surgery, Sports Traumatology, Arthroscopy*.

[28] Jo C. H., Lee Y. G., Shin W. H. (2014). Intra-articular injection of mesenchymal stem cells for the treatment of osteoarthritis of the knee: a proof-of-concept clinical trial. *STEM CELLS*.

[29] Roelofs A. J., Rocke J. P. J., De Bari C. (2013). Cell-based approaches to joint surface repair: a research perspective. *Osteoarthritis and Cartilage*.

[30] Murphy M. B., Moncivais K., Caplan A. I. (2013). Mesenchymal stem cells: environmentally responsive therapeutics for regenerative medicine. *Experimental and Molecular Medicine*.

[31] Sánchez M., Delgado D., Sánchez P. (2016). Combination of intra-articular and intraosseous injections of platelet rich plasma for severe knee osteoarthritis: a pilot study. *BioMed Research International*.

[32] Sánchez M., Fiz N., Guadilla J. (2014). Intraosseous infiltration of platelet-rich plasma for severe knee osteoarthritis. *Arthroscopy Techniques*.

[33] Muinos-López E., Ripalda-Cemboráin P., López-Martínez T. (2016). Hypoxia and reactive oxygen species homeostasis in mesenchymal progenitor cells define a molecular mechanism for fracture nonunion. *STEM CELLS*.

[34] Jones E. A., English A., Henshaw K. (2004). Enumeration and phenotypic characterization of synovial fluid multipotential mesenchymal progenitor cells in inflammatory and degenerative arthritis. *Arthritis & Rheumatism*.

[35] Renn T.-Y., Kao Y.-H., Wang C.-C., Burnouf T. (2015). Anti-inflammatory effects of platelet biomaterials in a macrophage cellular model. *Vox Sanguinis*.

[36] Vasina E. M., Cauwenberghs S., Feijge M. A., Heemskerk J. W., Weber C., Koenen R. R. (2011). Microparticles from apoptotic platelets promote resident macrophage differentiation. *Cell Death & Disease*.

[37] Fahy N., Farrell E., Ritter T., Ryan A. E., Murphy J. M. (2015). Immune modulation to improve tissue engineering outcomes for cartilage repair in the osteoarthritic joint. *Tissue Engineering Part B: Reviews*.

[38] Jiang Y., Tuan R. S. (2015). Origin and function of cartilage stem/progenitor cells in osteoarthritis. *Nature Reviews Rheumatology*.

[39] Krüger J. P., Endres M., Neumann K. (2012). Chondrogenic differentiation of human subchondral progenitor cells is affected by synovial fluid from donors with osteoarthritis or rheumatoid arthritis. *Journal of Orthopaedic Surgery and Research*.

[40] Fahy N., de Vries-van Melle M. L., Lehmann J. (2014). Human osteoarthritic synovium impacts chondrogenic differentiation of mesenchymal stem cells via macrophage polarisation state. *Osteoarthritis and Cartilage*.

[41] Pretzel D., Linss S., Rochler S. (2011). Relative percentage and zonal distribution of mesenchymal progenitor cells in human osteoarthritic and normal cartilage. *Arthritis Research and Therapy*.

[42] Endres M., Neumann K., Häupl T. (2007). Synovial fluid recruits human mesenchymal progenitors from subchondral spongious bone marrow. *Journal of Orthopaedic Research*.

[43] Assirelli E., Filardo G., Mariani E. (2014). Effect of two different preparations of platelet-rich plasma on synoviocytes. *The Knee Surgery, Sports Traumatology, Arthroscopy*.

[44] Yuan X. L., Meng H. Y., Wang Y. C. (2014). Bone-cartilage interface crosstalk in osteoarthritis: potential pathways and future therapeutic strategies. *Osteoarthritis and Cartilage*.

[45] Lee C. H., Cook J. L., Mendelson A., Moioli E. K., Yao H., Mao J. J. (2010). Regeneration of the articular surface of the rabbit synovial joint by cell homing: A Proof of Concept Study. *The Lancet*.

[46] Anitua E., Prado R., Azkargorta M. (2015). High-throughput proteomic characterization of plasma rich in growth factors (PRGF-Endoret)-derived fibrin clot interactome. *Journal of Tissue Engineering and Regenerative Medicine*.

[47] Kulawig R., Krüger J. P., Klein O. (2013). Identification of fibronectin as a major factor in human serum to recruit subchondral mesenchymal progenitor cells. *The International Journal of Biochemistry & Cell Biology*.

[48] Rice J. J., Martino M. M., De Laporte L., Tortelli F., Briquez P. S., Hubbell J. A. (2013). Engineering the regenerative microenvironment with biomaterials. *Advanced Healthcare Materials*.

[49] Arufe M. C., De La Fuente A., Fuentes I., De Toro F. J., Blanco F. J. (2010). Chondrogenic potential of subpopulations of cells expressing mesenchymal stem cell markers derived from human synovial membranes. *Journal of Cellular Biochemistry*.

[50] Iwakura T., Sakata R., Reddi A. H. (2013). Induction of chondrogenesis and expression of superficial zone protein in synovial explants with TGF-*β*1 and BMP-7. *Tissue Engineering Part A*.

[51] Mak J., Jablonski C. L., Leonard C. A. (2016). Intra-articular injection of synovial mesenchymal stem cells improves cartilage repair in a mouse injury model. *Scientific Reports*.

[52] Lee J.-C., Min H. J., Park H. J., Lee S., Seong S. C., Lee M. C. (2013). Synovial membrane-derived mesenchymal stem cells supported by platelet-rich plasma can repair osteochondral defects in a rabbit model. *Arthroscopy: The Journal of Arthroscopy & Related Surgery*.

